# Correlation between hemoglobin and the risk of common malignant tumors: a 1999–2020 retrospective analysis and causal association analysis

**DOI:** 10.1186/s12885-024-12495-0

**Published:** 2024-06-21

**Authors:** Guo-Sheng Li, Tao Huang, Jing-Xiao Li, Jun Liu, Xiang Gao, Nuo Yang, Hua-Fu Zhou

**Affiliations:** 1 Department of Cardiothoracic Surgery, The First Affiliated Hospital of Guangxi Medical University, Guangxi Zhuang Autonomous Region, Nanning, 530021 P. R. China; 2 Department of Cardiothoracic Vascular Surgery, The Affiliated Hospital of Youjiang Medical University for Nationalities, Guangxi Zhuang Autonomous Region, Baise, 533099 P. R. China

**Keywords:** Cancer, Risk factor, Biomarker, Mendelian randomization analysis

## Abstract

**Background:**

The role of hemoglobin (HGB) in common malignant tumors remains unclear.

**Methods:**

A retrospective analysis was conducted to identify the correlation between HGB levels and risk of 15 malignant tumors using 50,085 samples from the National Health and Nutrition Examination Survey. Mendelian Randomization analyses (MRAs) were performed based on genome-wide association study data to assess the causal relationship between HGB levels and these malignant tumors using more than 700,000 samples. The robustness of the MRA results was confirmed through various analytical methods. Fifty-six in-house samples were used to investigate the correlation between HGB levels and the prognosis in prostate cancer (PRCA) using the Kaplan-Meier curve.

**Results:**

High HGB levels were associated with a higher risk for patients with cervix cancer, melanoma, and non-melanoma skin cancer (OR > 1.000, *p* < 0.05). It served as a protective factor for colon cancer, esophagus cancer, stomach cancer, bone cancer, lung cancer, renal cancer, and PRCA (OR < 1.000, *p* < 0.05). Furthermore, MRAs suggested that elevated HGB levels were correlated with a reduced risk of PRCA (OR = 0.869, *p* < 0.05), with no significant association observed between this marker and the remaining 14 malignant tumors. No pleiotropy or heterogeneity was found in the ultimate results for MRAs (*p*-values > 0.05), suggesting the robustness of the results. The results derived from the in-house data revealed a relationship between higher HGB values and a more favorable prognosis in PRCA (*p* < 0.05).

**Conclusion:**

High circulating HGB levels may play a protective prognostic role for PRCA and serve as a protective factor against the occurrence of PRCA.

**Supplementary Information:**

The online version contains supplementary material available at 10.1186/s12885-024-12495-0.

## Introduction

Tumors pose a significant global health concern and are among the leading causes of death worldwide, second only to cardiovascular diseases. Each year, millions of people receive a tumor diagnosis, and millions more succumb to this disease [1]. While prognosis varies across different types of tumors, patients diagnosed in later stages generally have a poorer prognosis than those diagnosed at early stages [2–4]. Further, clinical approaches such as surgical interventions offer more apparent benefits for early-stage tumor patients [2–4]. Therefore, early identification of potential tumor patients is crucial, making the exploration of biomarkers particularly important.

Among the various clinical testing methods available, the blood routine test is increasingly garnering the attention of healthcare professionals due to its advantages of simplicity, rapid execution, and cost-effectiveness. Recent studies have indicated that alterations in certain common indicators in the blood routine test might serve as causative factors in tumor development, with their irregularities potentially aiding in the identification of individuals at risk of cancer. For instance, in comparison to healthy control groups, patients diagnosed with colorectal cancer may exhibit elevated levels of eosinophils and lymphocytes [5]. The increase in elevated eosinophil and lymphocyte levels is directly correlated with a heightened susceptibility to colorectal cancer [5]. Likewise, a rise in platelet count has been associated with an increased lung cancer risk [6]. Individuals with high hemoglobin (HGB) levels are less likely to develop bladder cancer (BLCA), with high HGB serving as a protective prognostic factor [7, 8]. These research findings underscore the significant importance of hematological indicators, such as HGB, in the early detection of tumors. Nevertheless, there exists a gap in current research that investigates the specific correlation between changes in hematological indicators and different types of human tumors. Furthermore, existing studies predominantly concentrate on exploring the connection between various types of white blood cells and cancer patients, while the understanding of the relationship between HGB and tumors remains limited. This underscores the necessity for further research in this particular domain.

This study aims to explore the association between HGB and 15 common human malignancies through a retrospective analysis and a Mendelian randomization analysis (MRA). Firstly, we conducted a preliminary exploration of the potential association between HGB and the 15 malignancies using large-scale data from the National Health and Nutrition Examination Survey (NHANES). MRA is a method that utilizes instrumental variables (IVs), particularly single nucleotide polymorphisms (SNPs), to assess whether an exposure factor influences specific outcomes. Therefore, by utilizing data from genome-wide association studies (GWAS) and the MRA approach, we analyzed the causal association between HGB and these malignancies.

## Materials and methods

### Study design

**Fig. 1:**
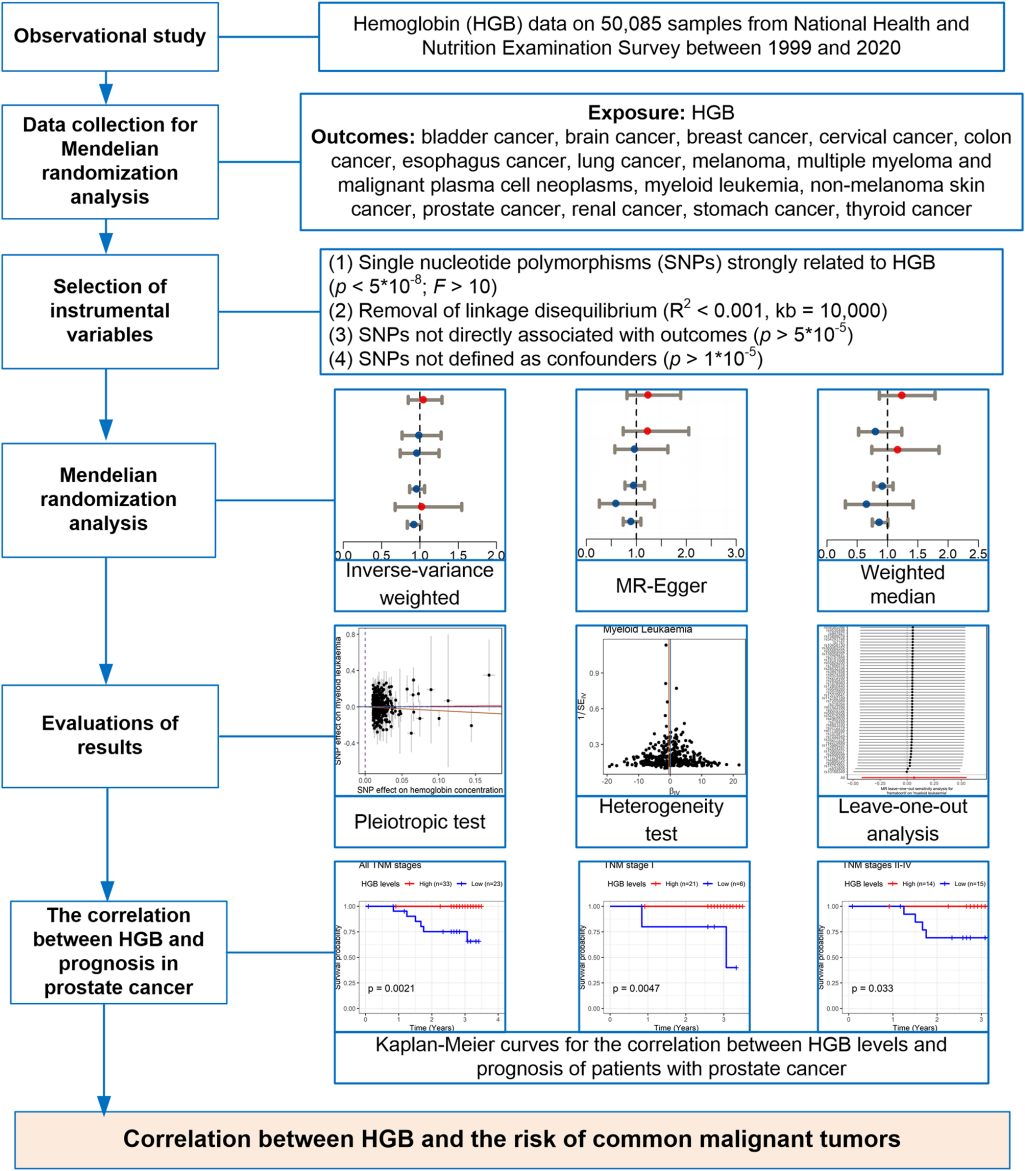
An overview of this study

This study aims to investigate the causal relationship between HGB levels and the 15 most common types of malignant tumors in humans (Fig. 1). These tumors collectively account for approximately 70% of all human tumors, including the most prevalent malignancies across most of eight biological systems [9]. The study is divided into three parts. The first part involves an observational study that utilizes data from the NHANES to assess the association between HGB levels and malignant tumors. In the second part, this study explores the causal relationship between HGB levels and the risk of developing the 15 types of malignant tumors using GWAS data. Several key questions need to be clarified: (1) whether the IVs used for MRA affect HGB, thereby influencing the occurrence of malignant tumors; (2) whether these IVs are unrelated to known confounding factors associated with malignant tumors; (3) whether the IVs have no direct relationship with the risk of malignant tumors [10]. The third part is the investigation of the correlation between HGB levels and the prognosis of patients with prostate cancer (PRCA) using an in-house cohort. The study was approved by the Ethics Committee of the First Affiliated Hospital of Guangxi Medical University (GXMU-FAH) (No. 2023-E605-01).

### Data collection for observational study

For the observational study section, we employed the NHANES database, a nationally representative survey of the United States population. This database comprises an interview conducted at the participant’s residence, along with pertinent information on demographics, clinical examinations, and laboratory results. Our study included only individuals who met the inclusion criteria: age of 20 years or older, participation in the survey between 1999 and 2020, and provision of definitive information on their cancer status. We collected data on variables such as HGB levels, cancer status, and demographics, including age and gender.

### Collection of GWAS data

This study utilized data downloaded from the FinnGen database in the IEU Open GWAS project (https://gwas.mrcieu.ac.uk/) to investigate the causal relationship between HGB levels and the risk of malignant tumors using MRAs. The exposure cohort used in this study is labeled as “ebi-a-GCST90002310” [11], which comprises 563,946 samples and includes 46,755,983 SNPs from European populations. Fifteen outcome cohorts, all consisting of European populations, were examined to correspond with 15 distinct types of malignant tumors. These include: (1) myeloid leukemia (related to the circulatory system); (2) colon cancer, esophageal cancer, and stomach cancer (associated with the digestive system); (3) thyroid cancer (pertaining to the endocrine system), (4) multiple myeloma and malignant plasma cell neoplasms (related to the motor system); (5) brain cancer (associated with the nervous system); (6) cervix cancer and PRCA (for the reproductive system); (7) lung cancer (related to the respiratory system); (8) BLCA and renal cancer (concerning the urinary system); (9) breast cancer, melanoma, and non-melanoma skin cancer (from other sources).

### Selection of IVs

SNPs closely associated with HGB were carefully selected as IVs for MRAs. The IVs chosen for this study met the following criteria: First, SNPs had to show a significant association with HGB, with a *p*-value threshold of < 5*10^− 8^. Second, the selected SNPs had to show a strong correlation with HGB, indicated by an *F*-test value higher than 10. Third, SNPs had to be randomly distributed and not in linkage disequilibrium, ensuring an R^2^ value of less than 0.001 within a 10,000-kb clumping distance. Fourth, IVs had to have no direct relationship with the outcome variable, namely the status of malignant tumors, with a *p*-value greater than 5*10^− 5^. Fifth, IVs associated with confounding factors contributing to common cancers were excluded based on a Phenoscanner [12] database-identified *p*-value lower than 1*10^− 05^.

### MRAs and their evaluation methods

Three MRA methods were used to explore the potential causal relationship between HGB and cancer risk: inverse variance-weighted (IVW), Mendelian randomization-Egger (MR-Egger), and weighted median (WM) methods. IVW was the primary method for evaluating the results, while MR-Egger and WM were used to assess the robustness of the IVW results. HGB may be considered a risk or protective factor for cancer occurrence if the IVW analysis results are statistically significant and trends observed from the other two methods are consistent with IVW.

In order to assess the robustness of the MRA results, a variety of methods were employed. Scatter plots and Egger regression statistical test were utilized to evaluate potential horizontal pleiotropy. Additionally, funnel plots and Cochran’s Q statistic test were employed to identify any heterogeneity in the SNP data. Furthermore, leave-one-out analysis was conducted to determine whether individual SNPs impacted the MRA effect. If a cancer exhibited unstable results in the leave-one-out analysis, additional cohorts sourced from the IEU Open GWAS project were collected and utilized to perform replicated MRAs for those specific cancers, employing the same processes outlined above.

### Collection of in-house data

An in-house GXMU-FAH cohort was collected to investigate the correlation between HGB levels and the prognosis of patients with PRCA. The inclusion criteria for the cohort were as follows: (1) patients admitted to GXMU-FAH between November 1, 2020, and October 31, 2021; (2) patients undergoing their initial surgery for PRCA at GXMU-FAH; (3) pathological confirmation of PRCA in the surgical specimen; (4) Availability of complete TNM staging (the eighth edition) information. Exclusion criteria were: (1) previous treatment for PRCA, including chemotherapy; (2) age under 20 years old.

### Other statistical analysis

Baseline information of NHANES samples was plotted using the *Tableone* package in R (version 4.1.0). Logistic regression was used to analyze the correlation between HGB and the 15 types of malignant tumors in NHANES data. For the GXMU-FAH cohort, Kaplan-Meier curves and log-rank tests were employed to investigate the correlation between HGB levels and the prognosis of patients across various TNM stages. The grouping threshold for the high-HGB and low-HGB groups was determined by the *survminer* package using maximally selected rank statistics. All analyses in this study were performed using R (version 4.1.0) and relevant packages, including the *TwoSampleMR* [13], *dplyr*, *survival*, and *survminer* packages.

## Results

### Data used in this study

Three sources of data were used for this study. In detail, 3551 malignant tumor samples and 46,534 healthy individual samples were collected from NHANES for observational analysis. As shown in Table 1, the mean age of these individuals from the NHANES ranged from 47.24 to 73.74 years old, with most of them being females. As illustrated in Supplementary Material 1, the study included 35,850 cancer cases and more than 200,000 control individuals from the IEU Open GWAS project for the original MRAs. Furthermore, an additional four cohorts, comprising 6606 cancer cases and more than 460,000 controls, were collected from the IEU Open GWAS for the replicated MRAs. Fifth-six samples were obtained from GXMU-FAH and were utilized to investigate the relationship between HGB levels and prognosis in PRCA.

**Table 1:** The demographic data of cohorts from National Health and Nutrition Examination Survey

Category	Age (mean ± SD), years	Female (%)	Male (%)	HGB (mean ± SD), g/dL
Bladder cancer	73.74 ± 9.09	23 (21.50)	84 (78.50)	14.16 ± 1.80
Blood cancer	63.78 ± 16.24	3 (33.30)	6 (66.70)	13.97 ± 1.54
Bone cancer	61.38 ± 19.26	11 (42.30)	15 (57.70)	12.98 ± 2.19
Brain cancer	54.74 ± 17.68	9 (47.40)	10 (52.60)	14.05 ± 0.85
Breast cancer	67.48 ± 11.85	693 (99.90)	1 (0.10)	13.35 ± 1.26
Cervix cancer	47.24 ± 15.10	320 (100.00)	0 (0.00)	13.62 ± 1.30
Colon cancer	71.00 ± 11.28	137 (47.40)	152 (52.60)	13.70 ± 1.42
Esophagus cancer	68.50 ± 11.08	6 (30.00)	14 (70.00)	13.18 ± 2.13
Kidney cancer	68.79 ± 13.20	26 (30.20)	60 (69.80)	13.77 ± 1.66
Lung cancer	67.96 ± 11.69	48 (43.20)	63 (56.80)	13.65 ± 1.39
Melanoma cancer	66.27 ± 13.95	133 (44.90)	163 (55.10)	14.20 ± 1.44
Prostate cancer	72.62 ± 7.97	0 (0.00)	707 (100.00)	14.16 ± 1.53
Non-melanoma skin cancer	67.49 ± 13.23	323 (44.00)	411 (56.00)	14.24 ± 1.39
Stomach cancer	64.23 ± 14.18	20 (57.10)	15 (42.90)	13.02 ± 1.65
Thyroid cancer	55.87 ± 13.65	79 (80.60)	19 (19.40)	13.58 ± 1.29
Healthy control	48.11 ± 17.64	24,085 (51.8)	22,449 (48.2)	14.10 ± 1.57

### Correlation between HGB and 15 malignant tumors using NHANES data

A retrospective analysis was conducted using NHANES data. Table 2 presents the results, indicating that increasing age was found to be associated with an increased risk of 13 out of 15 types of malignant tumors (excluding brain cancer and cervix cancer), with a per 1-standard deviation (SD) odds ratio (OR) increase ranging from 1.02 to 1.13. These differences were statistically significant (*p* < 0.05). Male patients exhibited a higher risk of colon cancer, esophagus cancer, bone cancer, lung cancer, bladder cancer, and renal cancer, with a per 1-SD OR increase ranging from 1.35 to 3.97 (*p* < 0.05, Table 2). However, male patients had a lower risk of thyroid cancer and breast cancer, with a per 1-SD OR less than 1 (*p* < 0.05, Table 2).

Furthermore, high HGB levels were associated with a higher risk for patients with cervix cancer, melanoma, and non-melanoma skin cancer (per 1-SD OR increase = 1.12–1.29, Table 2). Conversely, it served as a protective factor for seven types of malignant tumors, including colon cancer, esophagus cancer, stomach cancer, bone cancer, lung cancer, renal cancer, and PRCA, with a per 1-SD OR ranging from 0.64 to 0.93 (*p* < 0.05, Table 2).

**Table 2:** The associations of hemoglobin with cancer risk using cohorts from National Health and Nutrition Examination Survey

System	Odds ratio (95% confidence interval)			
	Cancer	Age	Gender (male vs. female)	HGB
Circulatory system	Blood cancer	1.05 (1.01–1.10)^*^	2.46 (0.57–12.66)	0.90 (0.60–1.44)
Digestive system	Colon cancer	1.09 (1.08–1.11)^*^	1.35 (1.05–1.72)^*^	0.92 (0.85–0.99)^*^
	Esophagus cancer	1.07 (1.04–1.11)^*^	3.77 (1.47–10.90)^*^	0.69 (0.54–0.89)^*^
	Stomach cancer	1.05 (1.03–1.07)^*^	1.25 (0.61–2.52)	0.69 (0.57–0.85)^*^
Endocrine system	Thyroid cancer	1.02 (1.01–1.04)^*^	0.26 (0.15–0.44)^*^	0.99 (0.86–1.16)
Motor system	Bone cancer	1.04 (1.01–1.06)^*^	2.60 (1.15–6.07)^*^	0.64 (0.53–0.80)^*^
Nervous system	Brain cancer	1.02 (1.00–1.05)	1.25 (0.44–3.59)	0.96 (0.71–1.36)
Reproductive system	Cervix cancer	1.00 (0.99–1.00)	/	1.29 (1.17–1.41)^*^
	Prostate cancer	1.11 (1.10–1.12)^*^	/	0.93 (0.88–0.98)^*^
Respiratory system	Lung cancer	1.07 (1.06–1.09)^*^	1.71 (1.15–2.56)^*^	0.85 (0.76–0.97)^*^
Urinary system	Bladder cancer	1.13 (1.10–1.15)^*^	3.97 (2.49–6.56)^*^	1.04 (0.91–1.18)
	Renal cancer	1.08 (1.06–1.10)^*^	2.99 (1.86–4.93)^*^	0.85 (0.75–0.98)^*^
Others	Breast cancer	1.07 (1.07–1.08)^*^	< 0.01 (0.00–0.01)^*^	1.02 (0.96–1.08)
	Melanoma	1.07 (1.06–1.08)^*^	1.15 (0.89–1.49)	1.12 (1.03–1.22)^*^
	Non-melanoma skin cancer	1.08 (1.07–1.08)^*^	1.16 (0.99–1.37)	1.15 (1.09–1.21)^*^

*Note:* Odds ratio was calculated using the logistic regression analysis. ^*^p < 0.05

### Identification of the IVs

According to the established criteria mentioned earlier, IVs for MRAs were selected (Supplementary Material 2). Initially, 491 SNPs related to HGB were preliminarily screened based on their *p* values and R^2^ values. Subsequently, 310 SNPs that exhibited a strong correlation with HGB were identified, with an *F* value greater than 10. These 310 SNPs were then input into PhenoScanner, which revealed that 35 of them were associated with known confounding factors such as smoking, drinking, obesity, cancer, tumors, leukemia, multiple myeloma, lymphoma, and melanoma (Supplementary Material 3) [14–16]. Ultimately, 275 SNPs were identified to be used as IVs in the subsequent MRA.

### MRAs of HGB on cancer risk

**Fig. 2:**
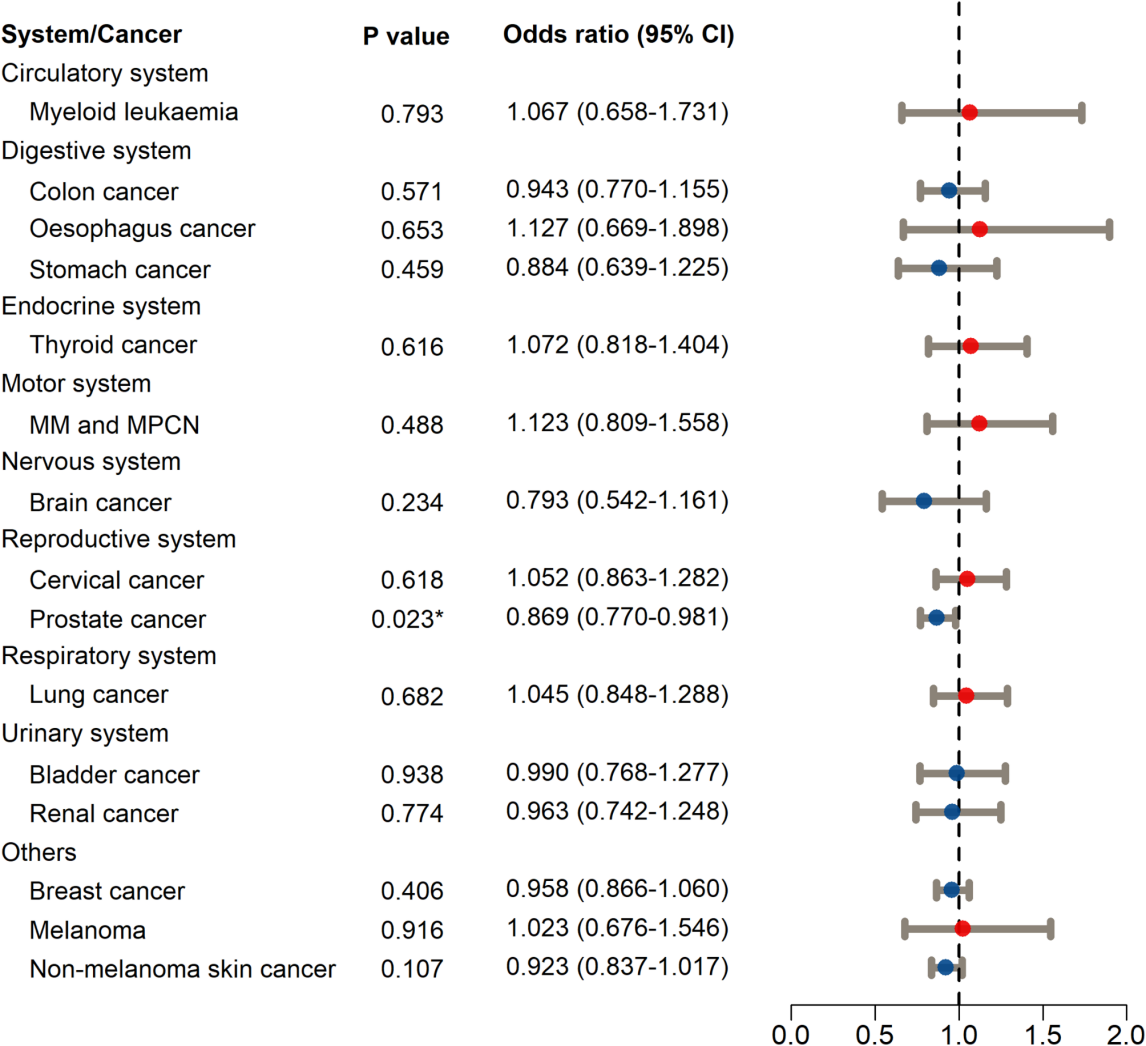
Associations of higher hemoglobin (HGB) with human cancers using Mendelian randomization analyses (inverse-variance weighted method). MM and MPCN, multiple myeloma and malignant plasma cell neoplasms. 95% CI, confidence interval. ^*^p < 0.05

The study utilized MRAs based on IVs to investigate causal associations between HGB levels and the risk of 15 types of malignant tumors from nine sources, including eight human biological systems and other sources. The primary MRA method (IVW) revealed that increased HGB was only associated with a reduced risk of PRCA in the reproductive system. Specifically, for each 1-SD increase in HGB, the risk of PRCA decreased by 13.1% (OR = 0.869, 95% CI: 0.770–0.981, *p* < 0.05, Fig. 2). This finding was further supported by the results of MR-Egger (Supplementary Material 4) and WM analyses (Supplementary Material 5), where the OR values were greater than 1. However, similar results were not observed for the other 14 types of malignant tumors (*p* > 0.05, Fig. 2).

### Evaluation of MRAs results

**Fig. 3:**
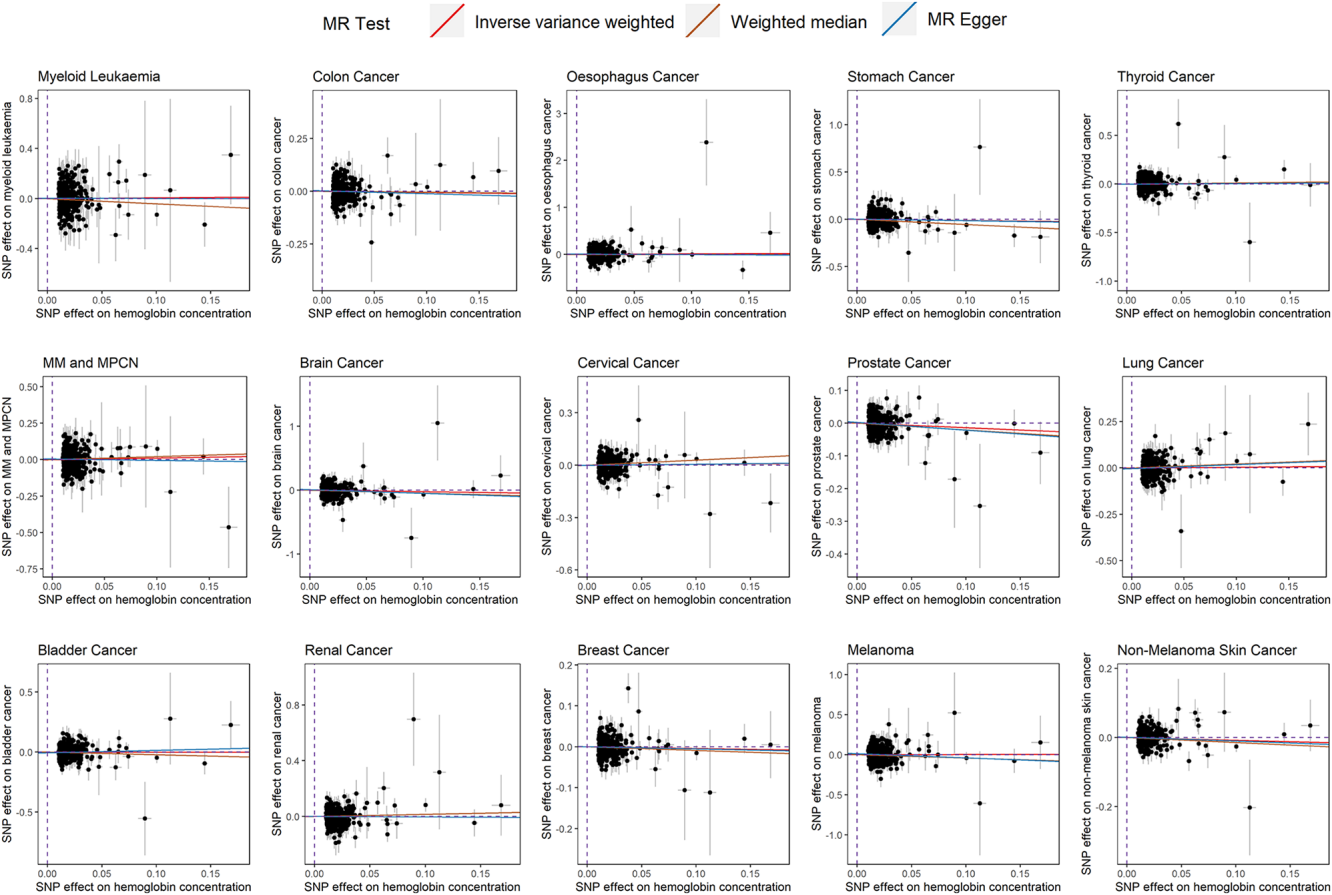
Scatter plots illustrating the effects of single nucleotide polymorphisms on hemoglobin and cancers

The study employed a series of methods to assess the robustness of MRAs results. The findings suggest that the scatter plot indicates a trend where the impact of SNPs on HGB tends towards 0, and their impact on 15 types of malignant tumors also tends towards 0 (Fig. 3), signifying no apparent evidence of detectable pleiotropy. Additionally, Egger regression statistical tests did not identify statistically significant pleiotropy (all *p*-values > 0.05; Supplementary Material 6), indicating an absence of pleiotropy in the effects of SNPs on both HGB and 15 types of cancer.

**Fig. 4:**
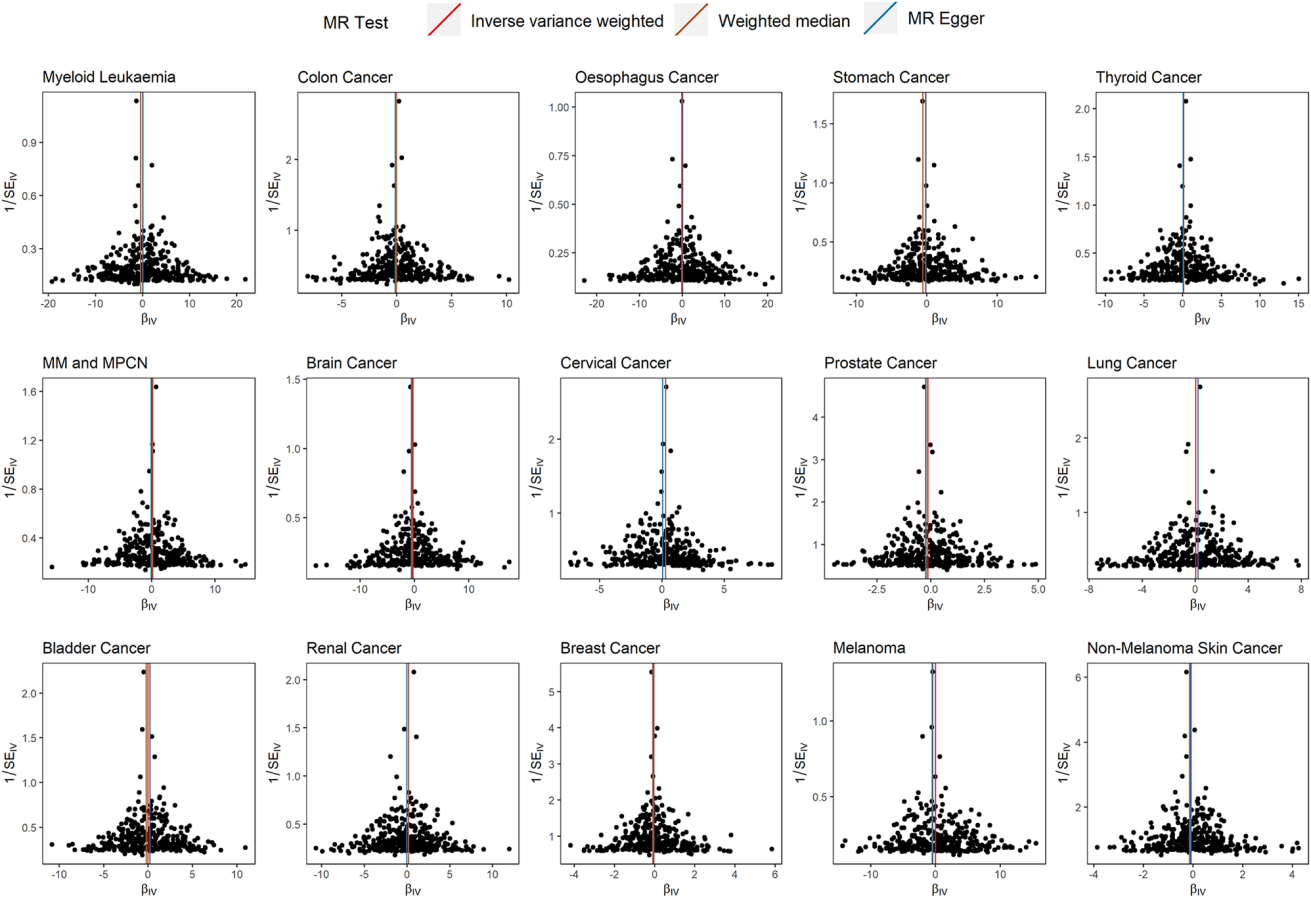
Funnel plots for detecting heterogeneity among single nucleotide polymorphisms for Mendelian randomization analyses

Figure 4 displays a symmetric distribution of the SNPs involved in the analysis of 15 types of malignant tumors in the funnel plot, suggesting no evident heterogeneity among these SNPs. Furthermore, an objective Cochran Q test revealed no heterogeneity among the SNPs in the MRAs of 14 types of malignant tumors (*p* > 0.05), except for stomach cancer (Supplementary Material 7). Although significant heterogeneity was observed among the SNPs related to stomach cancer (*p* < 0.05; Supplementary Material 7), the effect of heterogeneity on the conclusions based on the IVW method is limited [17], indicating the reliability of the MRAs results for stomach cancer.

The leave-one-out analysis results, as demonstrated in Supplementary Material 8, suggest that individual SNPs had no significant impact on the causal relationship between HGB and 11 types of malignant tumors (such as PRCA). However, some SNPs (19 for BLCA, 11 for melanoma, 1 for myeloid leukemia, and 1 for renal cancer) may affect the causal relationship between HGB and the risk of four types of malignant tumors (Supplementary Material 8). Therefore, the current findings for the four cancers need to be interpreted with caution. To address this issue, further MRAs were conducted based on additional data.

### Repeated MRAs on the effect of HGB on four cancers

**Fig. 5:**
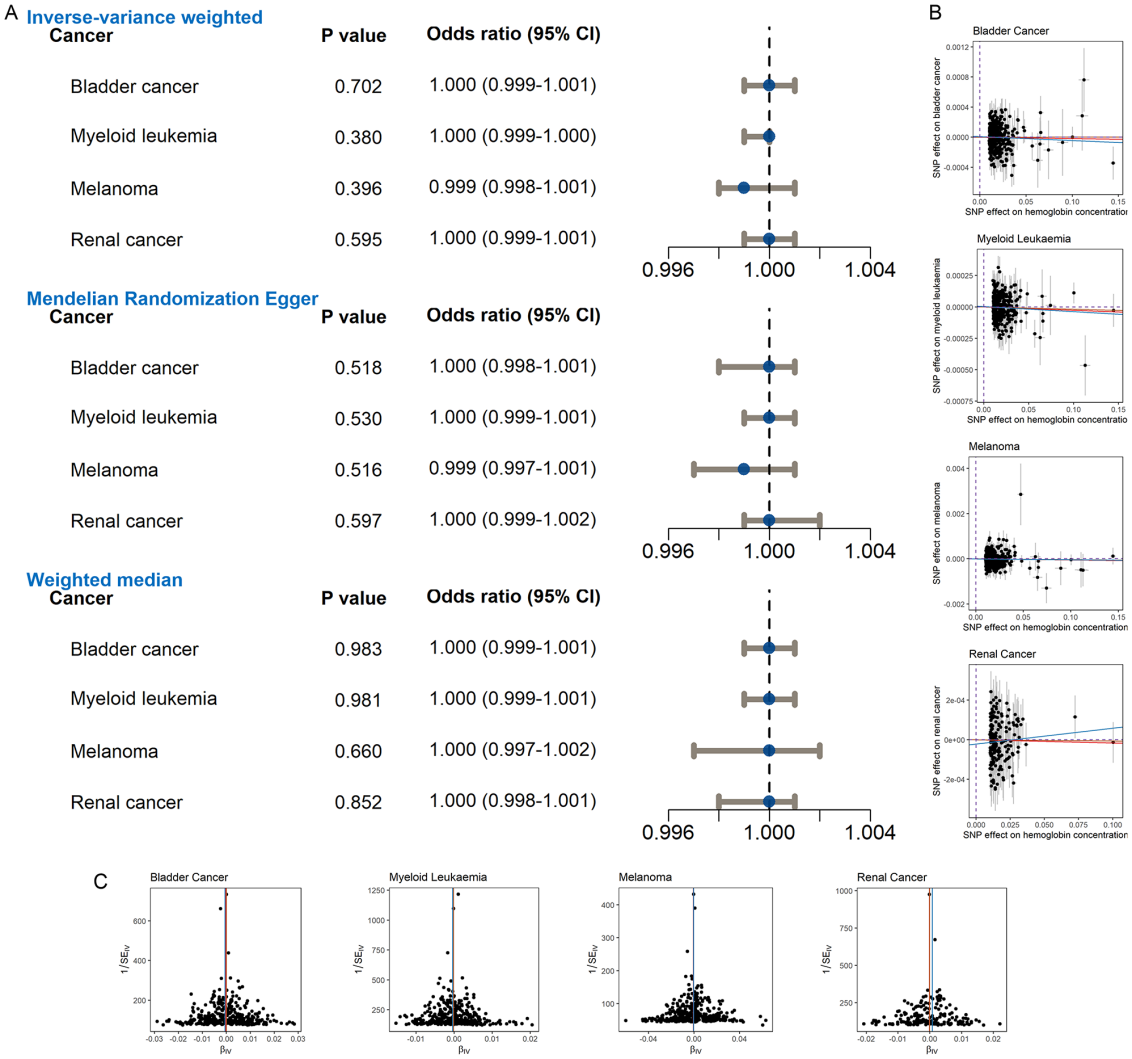
Investigation of the effect of HGB on specific cancers using four additional independent cohorts. Panel **A**: Evaluation of causal associations of higher HGB levels with cancers using Mendelian randomization analyses. Panel **B**: Scatter plots illustrating the effects of single nucleotide polymorphisms on HGB and cancers. Panel **C**: Funnel plots for detecting heterogeneity. 95% CI, confidence interval. ^*^p < 0.05

Using four additional cohorts (Supplementary Material 9) sourced from the IEU Open GWAS project, MRAs were replicated for BLCA, melanoma, myeloid leukemia, and renal cancer. The results indicated no significant causal relationship between HGB levels and the four types of cancer (*p* > 0.05, Fig. 5A). Neither the scatter plots (Fig. 5B) nor the Egger regression analyses (with *p*-values > 0.05, Supplementary Material 10) indicated significant pleiotropy. Additionally, funnel plots (Fig. 5C) and Cochran Q tests (*p* > 0.05, Supplementary Material 11) detected no heterogeneity in the MRA findings for BLCA, melanoma, myeloid leukemia, and renal cancer. As illustrated in Supplementary Material 12, none of the individual SNPs had a significant impact on the MRA results. Consequently, these findings do not identify the causal correlation between elevated HGB levels and an increased risk of BLCA, melanoma, myeloid leukemia, and renal cancer, which is consistent to the results shown in Fig. 2.

### Further investigation on the correlation between HGB and prognosis of PRCA

**Fig. 6:**
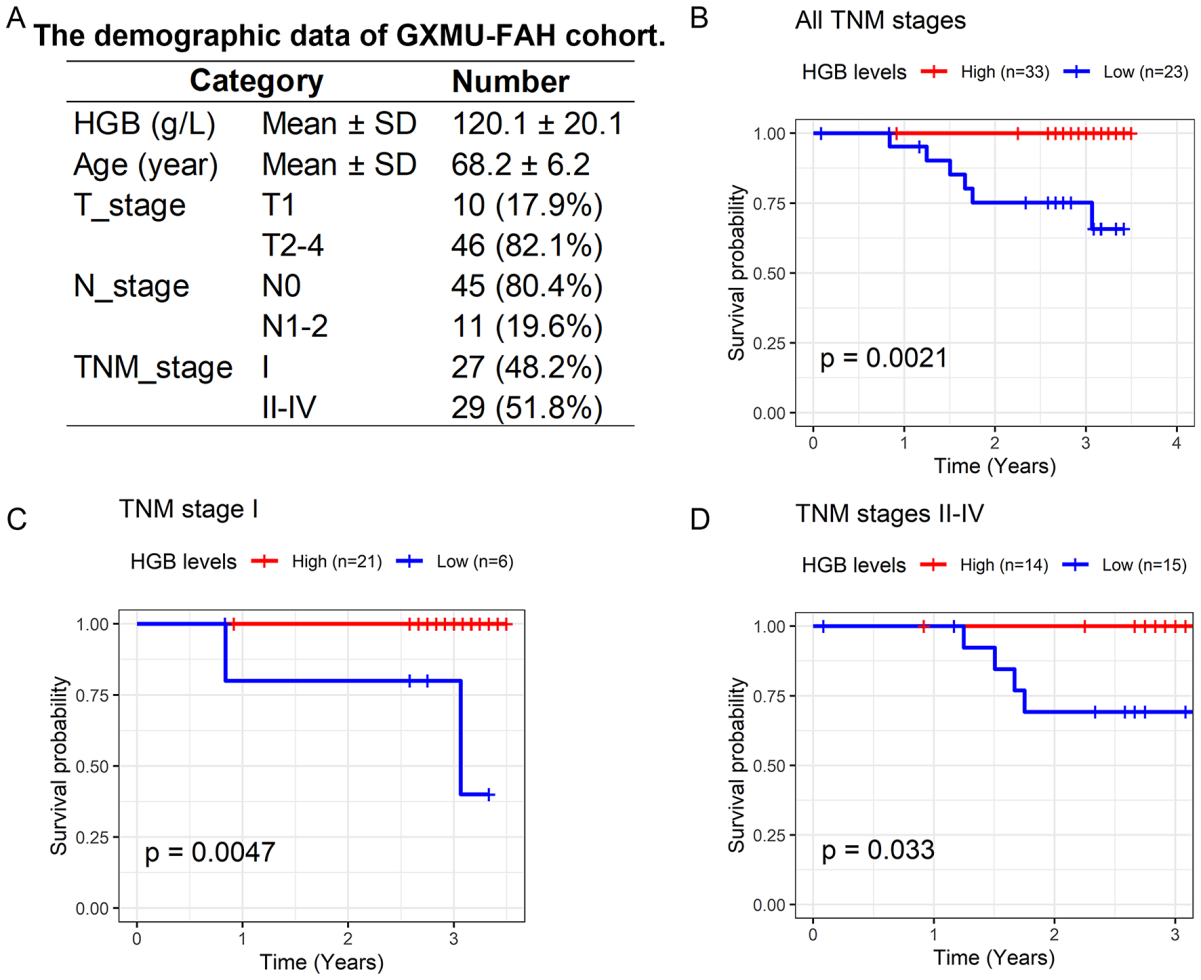
Investigation of the correlation between HGB levels and prognosis in PRCA. Panel **A**: Demographic data of the PRCA cohort from the First Affiliated Hospital of Guangxi Medical University. Panel **B**: Kaplan-Meier curve for the prognosis between the high HGB group and the low HGB group with stages I-IV; the grouping threshold for the low HGB group is < 116 g/L. Panel **C**: Kaplan-Meier curve for the prognosis between the high HGB group and the low HGB group with stage I; the grouping threshold for the low HGB group is < 105.5 g/L. Panel **D**: Kaplan-Meier curve for the prognosis between the high HGB group and the low HGB group with stages II-IV; the grouping threshold for the low HGB group is < 116 g/L. *P* values were calculated using log-rank tests

Recognizing HGB as a protective factor against PRCA occurrence, this study further investigated the correlation between HGB levels and the prognosis of patients with PRCA based on the in-house cohort. The GXMU-FAH cohort consisted of 56 male patients diagnosed with PRCA, aged between 49 and 83 years (Fig. 6A). Among them, 27 patients were at stage I, while the remainder were at stages II-IV (Fig. 6A). The Kaplan-Meier curve illustrates that patients in the high HGB group had significantly better overall survival than those in the low HGB group (*p* < 0.05, Fig. 6B). Stratified analysis indicated that both stage I and stages II-IV patients with PRCA in the high HGB group had a more favorable prognosis compared to those in the low HGB group (*p* < 0.05, Fig. 6C and D). Hence, HGB may function as a protective factor for the prognosis of patients with PRCA.

## Discussion

Tumors pose a significant threat to human health, making it imperative to investigate factors contributing to their onset and protection. Understanding these factors can inform the development of effective prevention and control strategies. This study aims to examine the association between HGB levels and 15 common malignant tumors originating from various human body systems and sources, using both retrospective analysis and MRAs. Initially, a retrospective analysis was conducted using a large sample (*n* = 50,085) from the NHANES database, revealing a linear correlation between HGB levels and nine specific malignant tumors. Subsequently, MRAs were performed based on GWAS data to assess the causal relationship between HGB levels and 15 malignant tumors. The findings indicate that HGB acts as a protective factor for PRCA. The robustness of these MRA results was confirmed through various analytical methods. Moreover, elevated HGB levels may function as a protective prognostic factor for patients with PRCA based on the in-house cohort. In summary, this study provides novel insights into the causal relationship between HGB levels and 15 common malignant tumors.

HGB appears to play diverse roles in different malignant tumors. Previous studies have suggested that decreased HGB levels are associated with an increased risk of tumors originating from various human body systems. For instance, Taylor et al. [18] demonstrated that preoperative anemia is linked to reduced overall survival in patients undergoing lung cancer surgery. Hakozaki et al. [19] identified low HGB levels (< 11 g/dL) as a prognostic risk factor for PRCA patients. Regarding BLCA, individuals with higher HGB levels are less likely to develop the disease, with high HGB serving as a protective factor in patient prognosis [7, 8]. Similarly, lower HGB levels were observed in patients with breast cancer compared to controls [20], and those patients with elevated HGB levels showed reduced tumor metastasis [21]. Moreover, our large-scale retrospective analysis validated that increased HGB levels may correlate with a decreased risk of lung cancer and PRCA. A similar trend was observed in five other cancers, including colon cancer, esophagus cancer, stomach cancer, bone cancer, and renal cancer. Conversely, elevated HGB levels were associated with an increased risk for patients with cervical cancer, melanoma, and non-melanoma skin cancer. These findings suggest that fluctuations in HGB levels may influence the risk of malignant tumors, contingent upon the specific tumor type. Notably, some of these findings in our study represent novel contributions to the existing literature, underscoring the significance of these results.

This study delves further into elucidating the causal relationship between HGB and 15 common malignant tumors. As previously mentioned, both earlier research and our retrospective analysis have indicated that fluctuations in HGB levels are associated with the risk of at least 10 malignant tumors. Notably, for PRCA, previous studies have reported the relationship between high HBG values and the favorable prognosis of this disease [19, 22, 23]. This is also verified in our analysis of the in-house data. In our study, further MRAs identified that high HGB levels are advantageous in reducing the risk of PRCA onset. These results indicate that high HGB levels can be used not only to assess the prognosis of patients with PRCA but also to reduce the risk of PRCA onset in the population. However, although MRAs suggest the causal relationship between HGB and the risk of PRCA occurrence, no similar association was observed between this marker and the remaining 14 malignant tumors. For instance, in the case of BLCA, previous research has highlighted the protective prognostic effect of high HGB levels, and a retrospective analysis has also suggested that individuals with elevated HGB levels have a lower susceptibility to BLCA [7, 8]. However, MRAs did not provide evidence to support the notion that elevated HGB levels were causative factors for BLCA. In contrast to retrospective analysis, MRAs are less susceptible to confounding factors and can elucidate the causal relationship between HGB levels and specific outcomes (such as PRCA), highlighting the importance of causal inference analysis.

The precise role of HGB in the progression of PRCA remains elusive. HGB, primarily found in red blood cells, plays a pivotal role in oxygen and carbon dioxide transport within the body. Aberrant alterations in HGB levels may be linked to tumor initiation and progression [24, 25]. On one hand, HGB deficiency can induce bodily hypoxia, leading to heightened oxygen radical generation, DNA damage, and genomic instability, thus fostering tumor development [26, 27]. On the other hand, HGB may impede blood flow and oxygen delivery by constricting blood vessel diameter, thereby diminishing nutrient supply to tumor tissues and impeding tumor cell proliferation [28, 29]. These attributes of HGB may confer an anti-cancer effect in tumors including PRCA. Regrettably, current literature lacks reports elucidating the role of HGB in the advancement of PRCA, underscoring the imperative for further in vivo and in vitro experiments to elucidate this phenomenon.

Several limitations are inherent to this study. Firstly, the MRA segment of this study exclusively relies on analyses of European populations, necessitating validation of these conclusions in other demographic cohorts in future investigations. Secondly, while this study posits a causal correlation between HGB levels and PRCA, the finding warrants validation through prospective studies. Additionally, HGB values may vary due to differences in testing institutions and equipment. Future prospective trials should adopt strict and standardized methods to measure HGB values in large populations. Considering that HGB levels may fluctuate over different time periods, measuring HGB levels of participants multiple times within a reasonable timeframe and calculating the average might be a more reliable strategy than a single measurement. Thirdly, although we were unable to perform animal experiments due to limited conditions, future efforts should aim to conduct in vivo and in vitro experiments to unravel the molecular mechanisms underpinning HGB function in PRCA and other cancers.

In summary, this study explores the association between HGB and various tumors, revealing its protective prognostic role in PRCA and its causal relationship with PRCA risk.

### Electronic supplementary material

Below is the link to the electronic supplementary material.

Supplementary Material 1

Supplementary Material 2

Supplementary Material 3

Supplementary Material 4

Supplementary Material 5

Supplementary Material 6

Supplementary Material 7

Supplementary Material 8

Supplementary Material 9

Supplementary Material 10

Supplementary Material 11

Supplementary Material 12

## Data Availability

The public data that support the findings of retrospective analysis and Mendelian Randomization analysis are available in National Health and Nutrition Examination Survey (https://www.cdc.gov/nchs/nhanes/index.htm) and IEU Open GWAS project (https://gwas.mrcieu.ac.uk/). The in-house data can be obtained from the corresponding authors upon request.
