# Supplementary material for: Correlation between hemoglobin and the risk of common malignant tumors: a 1999–2020 retrospective analysis and causal association analysis
Source: BMC Cancer. 2024 Jun 21;24:755. doi: 10.1186/s12885-024-12495-0 (PMC11193233; doi:10.1186/s12885-024-12495-0)
Supplement: Supplementary file 1 — Supplementary Material 1 [file 12885_2024_12495_MOESM1_ESM.pdf]

Supplementary material 1. Outcome cohorts for MRAs in this study.

System	Cancer type	Sample size (control vs. case)	Data source
Circulatory system	Myeloid leukemia	218509 vs. 283	finn-b-CD2_MYELOID_LEUKAEMIA
Digestive system	Colon cancer	216989 vs. 1803	finn-b-C3_COLON
	Esophagus cancer	218560 vs. 232	finn-b-C3_OESOPHAGUS
	Stomach cancer	218159 vs. 633	finn-b-C3_STOMACH
Endocrine system	Thyroid cancer	217803 vs. 989	finn-b-C3_THYROID_GLAND
Motor system	Multiple myeloma and malignant plasma cell neoplasms	218194 vs. 598	finn-b-CD2_MULTIPLE_MYELOMA_PLASMA_CELL
Nervous system	Brain cancer	218328 vs. 464	finn-b-C3_BRAIN
Reproductive system	Cervix cancer	121931 vs. 1648	finn-b-C3_CERVIX_UTERI
	Prostate cancer	88902 vs. 6311	finn-b-C3_PROSTATE
Respiratory system	Lung cancer	217165 vs. 1627	finn-b-C3_LUNG_NONSMALL
Urinary system	Bladder cancer	217677 vs. 1115	finn-b-C3_BLADDER
	Renal cancer	217821 vs. 971	finn-b-C3_KIDNEY_NOTRENALPELVIS
Others	Breast cancer	115178 vs. 8401	finn-b-C3_BREAST
	Melanoma	218399 vs. 393	finn-b-CD2_INSITU_MELANOMA
	Non-melanoma skin cancer	208410 vs. 10382	finn-b-C3_OTHER_SKIN

Notes: The data are available from https://gwas.mrcieu.ac.uk/datasets.
