# Supplementary material for: Correlation between hemoglobin and the risk of common malignant tumors: a 1999–2020 retrospective analysis and causal association analysis
Source: BMC Cancer. 2024 Jun 21;24:755. doi: 10.1186/s12885-024-12495-0 (PMC11193233; doi:10.1186/s12885-024-12495-0)
Supplement: Supplementary file 3 — Supplementary Material 3 [file 12885_2024_12495_MOESM3_ESM.pdf]

Supplementary material 3. The excluded IVs and their traits in this study.

SNP	Trait	PMID or other evidence
rs10423120	Breast cancer risk in BRCA1 mutation carriers	20852631
rs11556924	Number of self-reported non-cancer illnesses	UK Biobank
rs116219813	Current tobacco smoking	UK Biobank
rs12402990	Alcohol intake versus 10 years previously	UK Biobank
rs1294404	Waist circumference adjusted for smoking in females	28443625
rs12972151	Current tobacco smoking	UK Biobank
rs13107325	Body mass index in male non-smokers	28443625
rs13389219	Waist hip ratio in female non-smokers	28443625
rs165944	Alcohol usually taken with meals	UK Biobank
rs1716990	Multiple myeloma	22120009
rs17680229	High grade serous ovarian cancer	28346442
rs17709919	Number of self-reported non-cancer illnesses	UK Biobank
rs1800562	Alcohol consumption transferrin glycosylation	21665994
rs2294915	Alcoholic liver disease	UK Biobank
rs2561566	Number of cigarettes previously smoked daily	UK Biobank
rs28606370	Current tobacco smoking	UK Biobank
rs3104753	Self-reported breast cancer	UK Biobank
rs34862781	Number of self-reported non-cancer illnesses	UK Biobank
rs35106244	Alcohol intake frequency	UK Biobank
rs3785884	Alcohol intake frequency	UK Biobank
rs3791020	Alcohol usually taken with meals	UK Biobank
rs3845811	Alcohol usually taken with meals	UK Biobank
rs459193	Waist hip ratio in male non-smokers	28443625
rs4660253	Alcohol usually taken with meals	UK Biobank
rs4665390	Alcohol intake frequency	UK Biobank
rs550057	High grade serous ovarian cancer	28346442
rs62435145	Alcohol intake versus 10 years previously	UK Biobank
rs7137828	Ever smoked	UK Biobank
rs752590	Mucinous ovarian carcinoma	26075790
rs7526882	Light smokers, at least 100 smokes in lifetime	UK Biobank
rs7694958	Waist hip ratio in female non-smokers	28443625
rs7969559	Past tobacco smoking	UK Biobank
rs8077577	Obesity related traits	23251661
rs863678	Mucinous ovarian cancer	28346442
rs9429088	Body mass index adjusted for smoking in males	28443625
