# Supplementary material for: Correlation between hemoglobin and the risk of common malignant tumors: a 1999–2020 retrospective analysis and causal association analysis
Source: BMC Cancer. 2024 Jun 21;24:755. doi: 10.1186/s12885-024-12495-0 (PMC11193233; doi:10.1186/s12885-024-12495-0)
Supplement: Supplementary file 4 — Supplementary Material 4 [file 12885_2024_12495_MOESM4_ESM.pdf]

System/Cancer	P value	Odds ratio (95% CI)
Circulatory system		
Myeloid leukaemia	0.992	0.995 (0.375-2.641)
Digestive system		
Colon cancer	0.517	0.873 (0.580-1.315)
Oesophagus cancer	0.822	0.886 (0.309-2.538)
Stomach cancer	0.623	0.848 (0.439-1.637)
Endocrine system		
Thyroid cancer	0.838	1.059 (0.613-1.828)
Motor system		
MM and MPCN	0.778	0.909 (0.470-1.760)
Nervous system		
Brain cancer	0.143	0.562 (0.261-1.213)
Reproductive system		
Cervical cancer	0.751	1.067 (0.715-1.591)
Prostate cancer	0.057	0.789 (0.618-1.006)
Respiratory system		
Lung cancer	0.323	1.237 (0.812-1.887)
Urinary system		
Bladder cancer	0.435	1.227 (0.735-2.049)
Renal cancer	0.897	0.966 (0.572-1.632)
Others		
Breast cancer	0.606	0.948 (0.773-1.162)
Melanoma	0.219	0.593 (0.258-1.361)
Non-melanoma skin cancer	0.276	0.896 (0.735-1.091)

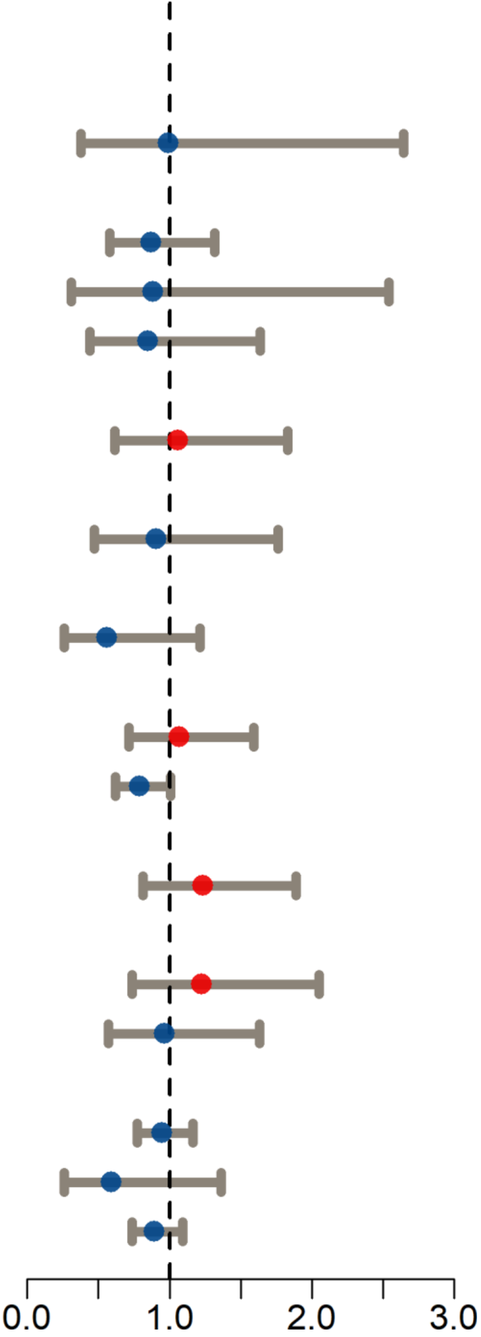

Supplementary material 4. Associations of higher HGB with human cancers using Mendelian randomization analysis (MR-Egger). 95% CI, confidence interval. $^{*}p < 0.05$.
