# Supplementary material for: Correlation between hemoglobin and the risk of common malignant tumors: a 1999–2020 retrospective analysis and causal association analysis
Source: BMC Cancer. 2024 Jun 21;24:755. doi: 10.1186/s12885-024-12495-0 (PMC11193233; doi:10.1186/s12885-024-12495-0)
Supplement: Supplementary file 5 — Supplementary Material 5 [file 12885_2024_12495_MOESM5_ESM.pdf]

System/Cancer	P value	Odds ratio (95% CI)
Circulatory system		
Myeloid leukaemia	0.332	0.665 (0.291-1.517)
Digestive system		
Colon cancer	0.808	0.959 (0.684-1.345)
Oesophagus cancer	0.940	0.964 (0.368-2.527)
Stomach cancer	0.065	0.575 (0.320-1.034)
Endocrine system		
Thyroid cancer	0.578	1.133 (0.731-1.756)
Motor system		
MM and MPCN	0.475	1.228 (0.699-2.158)
Nervous system		
Brain cancer	0.174	0.630 (0.323-1.227)
Reproductive system		
Cervical cancer	0.112	1.344 (0.933-1.934)
Prostate cancer	0.054	0.811 (0.656-1.003)
Respiratory system		
Lung cancer	0.244	1.240 (0.863-1.782)
Urinary system		
Bladder cancer	0.316	0.802 (0.522-1.234)
Renal cancer	0.503	1.170 (0.740-1.849)
Others		
Breast cancer	0.316	0.915 (0.769-1.089)
Melanoma	0.284	0.655 (0.302-1.421)
Non-melanoma skin cancer	0.057	0.866 (0.746-1.005)

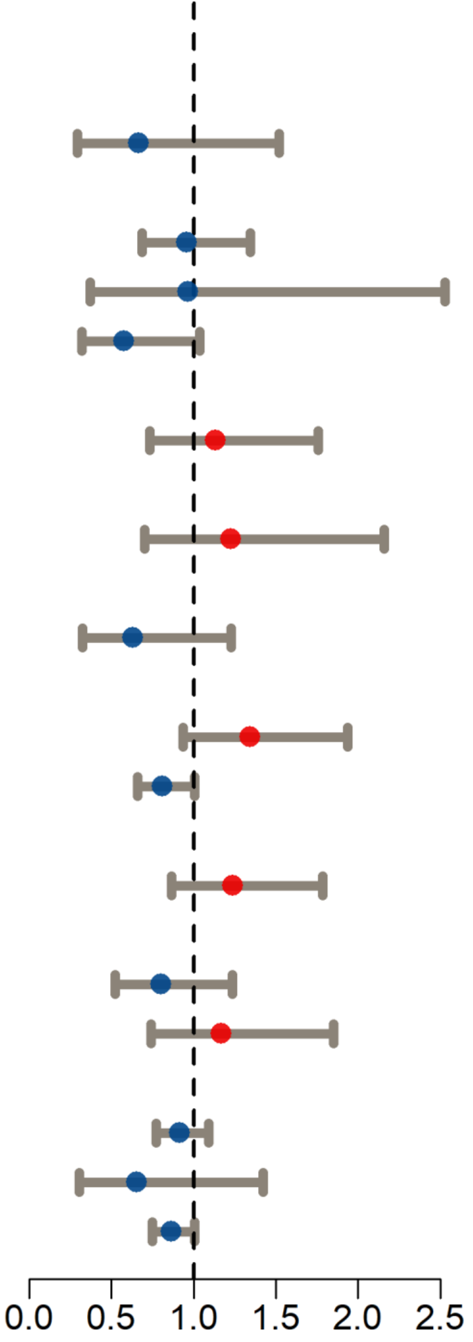

Supplementary material 5. Associations of higher HGB with human cancers using Mendelian randomization analysis (WM). 95% CI, confidence interval. $^{*}p < 0.05$.
