# Supplementary material for: Correlation between hemoglobin and the risk of common malignant tumors: a 1999–2020 retrospective analysis and causal association analysis
Source: BMC Cancer. 2024 Jun 21;24:755. doi: 10.1186/s12885-024-12495-0 (PMC11193233; doi:10.1186/s12885-024-12495-0)
Supplement: Supplementary file 6 — Supplementary Material 6 [file 12885_2024_12495_MOESM6_ESM.pdf]

Supplementary material 6. The Egger regression analysis for detecting horizontal pleiotropy.

Exposure	Cancer outcome	Egger intercept	SE	*P* value
Hemoglobin	Breast cancer	0.000	0.002	0.904
Hemoglobin	Bladder cancer	-0.005	0.006	0.345
Hemoglobin	Oesophagus cancer	0.006	0.012	0.607
Hemoglobin	Cervical cancer	0.000	0.004	0.935
Hemoglobin	Colon cancer	0.002	0.004	0.672
Hemoglobin	Stomach cancer	0.001	0.007	0.885
Hemoglobin	Lung cancer	-0.004	0.005	0.365
Hemoglobin	Brain cancer	0.008	0.008	0.313
Hemoglobin	Melanoma	0.013	0.009	0.140
Hemoglobin	Multiple myeloma and malignant plasma cell neoplasms	0.005	0.007	0.472
Hemoglobin	Myeloid leukaemia	0.002	0.011	0.872
Hemoglobin	Prostate cancer	0.002	0.003	0.367
Hemoglobin	Renal cancer	0.000	0.006	0.989
Hemoglobin	Non-melanoma skin cancer	0.001	0.002	0.736
Hemoglobin	Thyroid cancer	0.000	0.006	0.960
