# Supplementary material for: Correlation between hemoglobin and the risk of common malignant tumors: a 1999–2020 retrospective analysis and causal association analysis
Source: BMC Cancer. 2024 Jun 21;24:755. doi: 10.1186/s12885-024-12495-0 (PMC11193233; doi:10.1186/s12885-024-12495-0)
Supplement: Supplementary file 7 — Supplementary Material 7 [file 12885_2024_12495_MOESM7_ESM.pdf]

Supplementary material 7. The Cochran Q test for detecting heterogeneity.

Method	Exposure	Outcome	Cochran's Q value	*P* value
Inverse variance weighted	Hemoglobin	Breast cancer	85.345	0.530
Inverse variance weighted	Hemoglobin	Bladder cancer	90.627	0.374
Inverse variance weighted	Hemoglobin	Oesophagus cancer	67.590	0.939
Inverse variance weighted	Hemoglobin	Cervical cancer	70.240	0.905
Inverse variance weighted	Hemoglobin	Colon cancer	79.688	0.698
Inverse variance weighted	Hemoglobin	Stomach cancer	114.119	0.027
Inverse variance weighted	Hemoglobin	Lung cancer	104.554	0.097
Inverse variance weighted	Hemoglobin	Brain cancer	98.315	0.191
Inverse variance weighted	Hemoglobin	Melanoma	65.069	0.962
Inverse variance weighted	Hemoglobin	Multiple myeloma and malignant plasma cell neoplasms	76.292	0.787
Inverse variance weighted	Hemoglobin	Myeloid leukaemia	90.858	0.367
Inverse variance weighted	Hemoglobin	Prostate cancer	109.049	0.055
Inverse variance weighted	Hemoglobin	Renal cancer	88.692	0.429
Inverse variance weighted	Hemoglobin	Non-melanoma skin cancer	94.375	0.276
Inverse variance weighted	Hemoglobin	Thyroid cancer	102.847	0.118
