# Supplementary material for: Correlation between hemoglobin and the risk of common malignant tumors: a 1999–2020 retrospective analysis and causal association analysis
Source: BMC Cancer. 2024 Jun 21;24:755. doi: 10.1186/s12885-024-12495-0 (PMC11193233; doi:10.1186/s12885-024-12495-0)
Supplement: Supplementary file 8 — Supplementary Material 8 [file 12885_2024_12495_MOESM8_ESM.pdf]

Myeloid Leukaemia

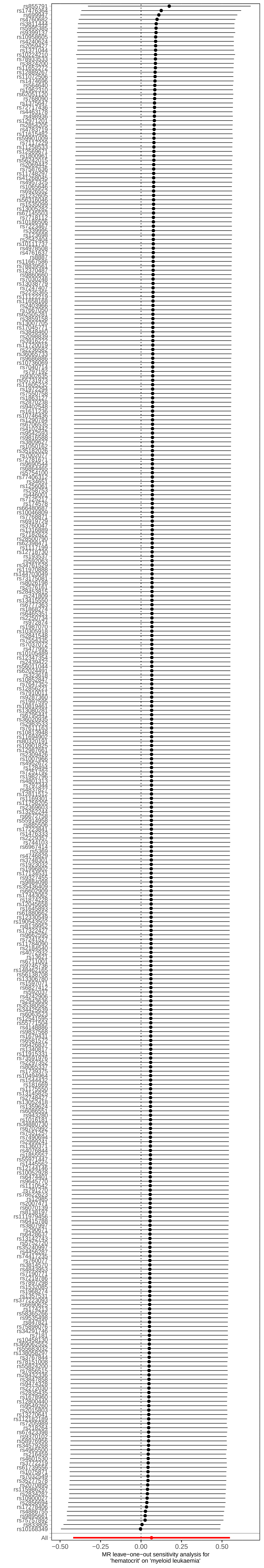

Colon Cancer

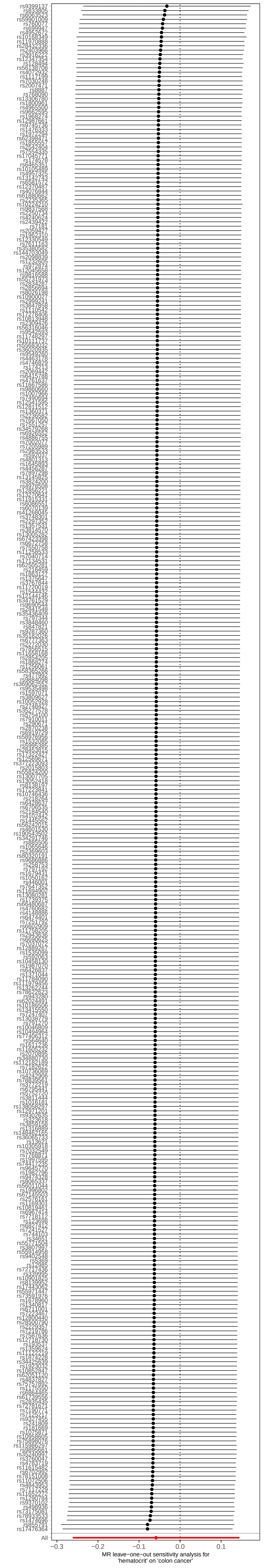

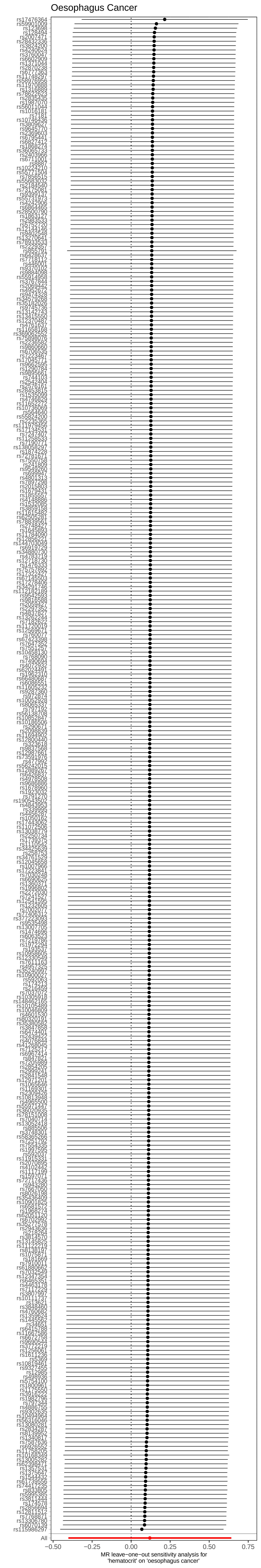

Oesophagus Cancer
All
-0.50
-0.25
0.00
0.25
0.50
0.75
MR leave-one-out sensitivity analysis for
'hematocrit' on 'oesophagus cancer'

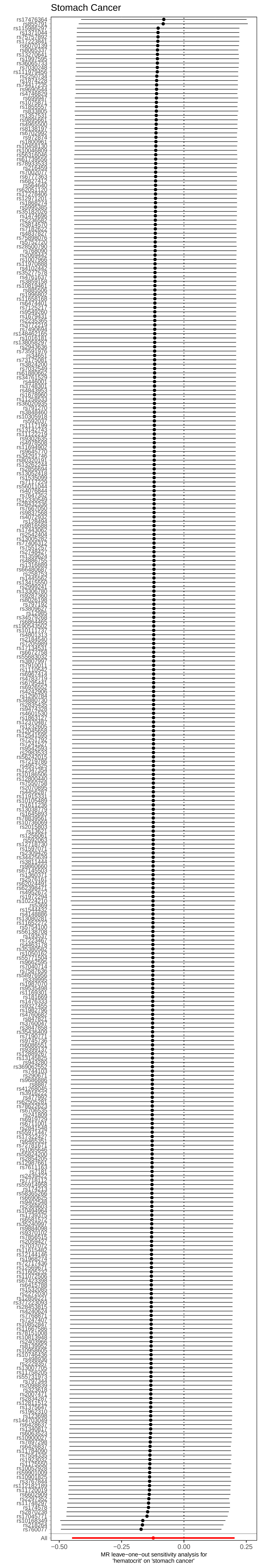

Stomach Cancer
All
-0.50
-0.25
0.00
0.25
MR leave−one−out sensitivity analysis for
'hematocrit' on 'stomach cancer'

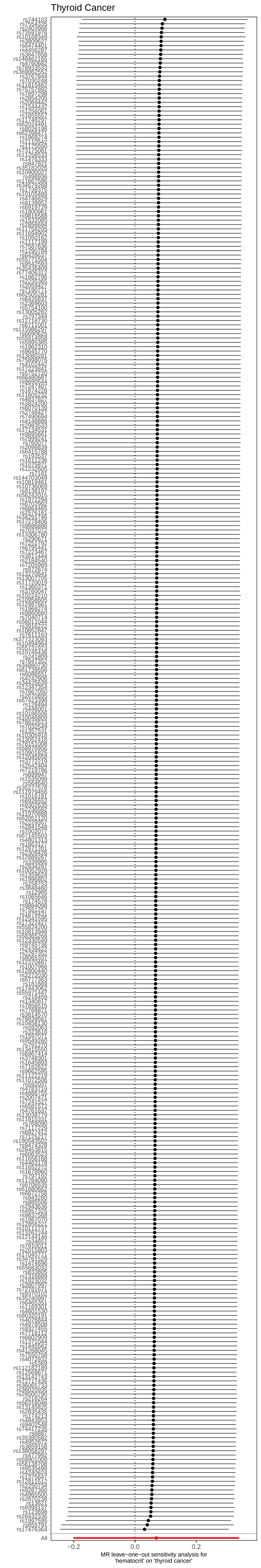
Thyroid Cancer
All
−0.2
0.0
0.2
MR leave−one−out sensitivity analysis for
'hematocrit' on 'thyroid cancer'

MM and MPCN

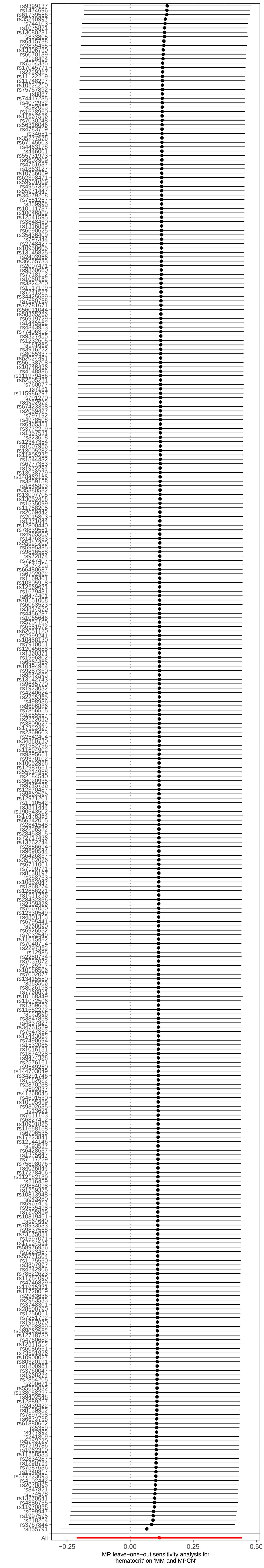
All
-0.25
0.00
0.25
0.50
MR leave−one−out sensitivity analysis for
'hematocrit' on 'MM and MPCN'

Brain Cancer

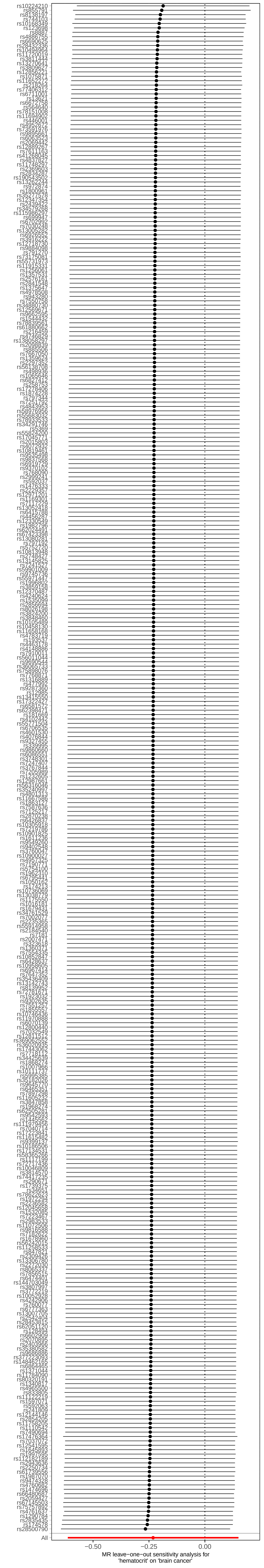

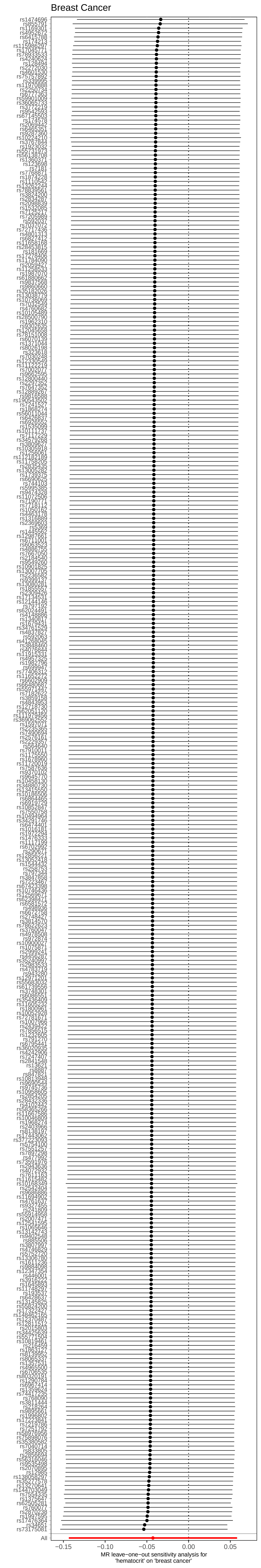
Breast Cancer
All
−0.15
−0.10
−0.05
0.00
0.05
MR leave−one−out sensitivity analysis for
'hematocrit' on 'breast cancer'

Melanoma

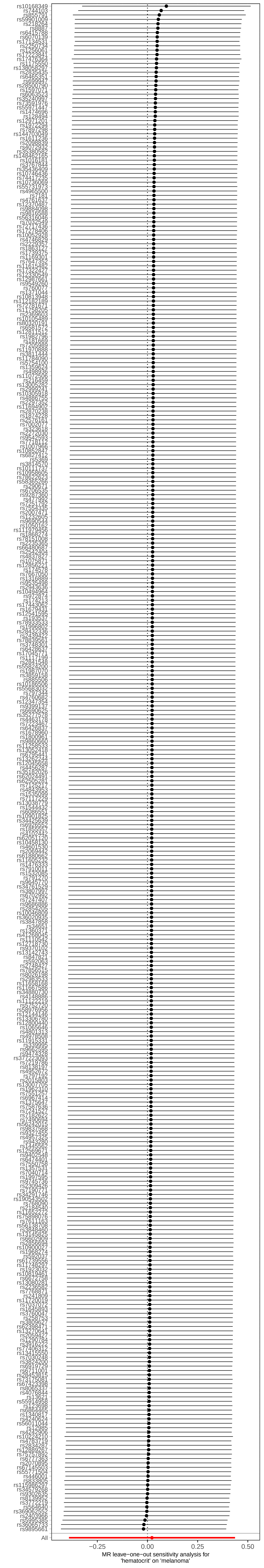

All
−0.25
0.00
0.25
0.50
MR leave−one−out sensitivity analysis for
'hematocrit' on 'melanoma'

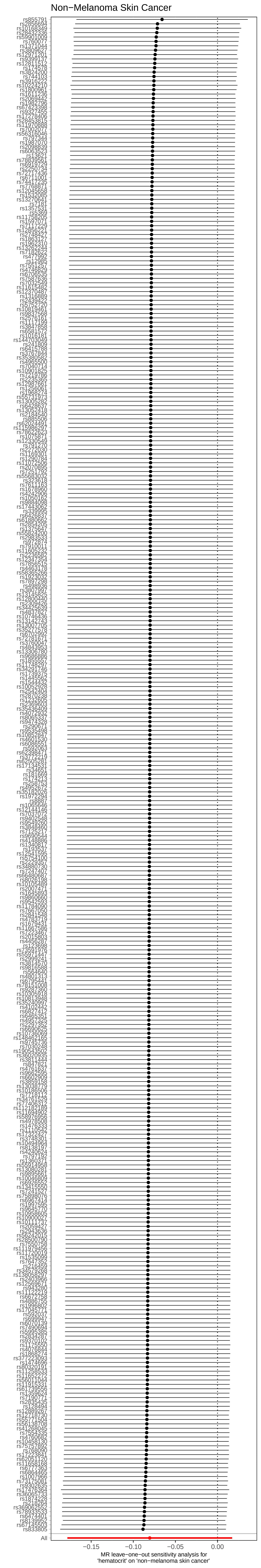

Non−Melanoma Skin Cancer
All
−0.15
−0.10
−0.05
0.00
MR leave−one−out sensitivity analysis for
'hematocrit' on 'non−melanoma skin cancer'

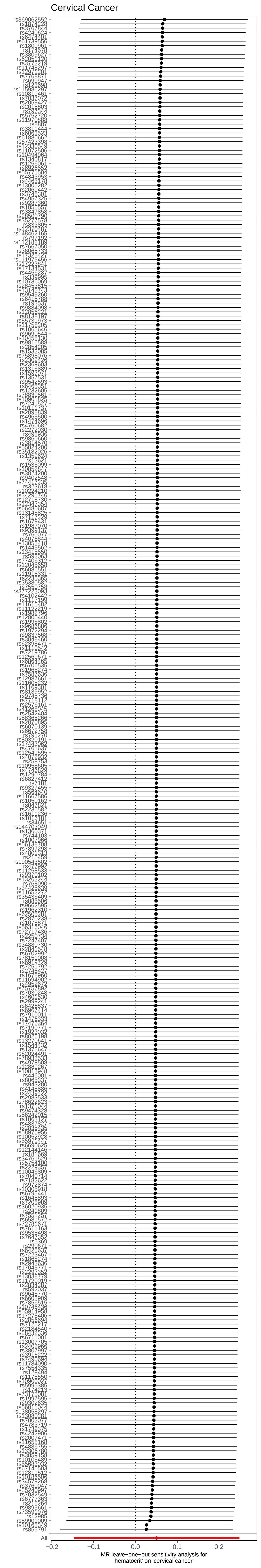
Cervical Cancer
All
−0.2
−0.1
0.0
0.1
0.2
MR leave−one−out sensitivity analysis for
'hematocrit' on 'cervical cancer'

Prostate Cancer

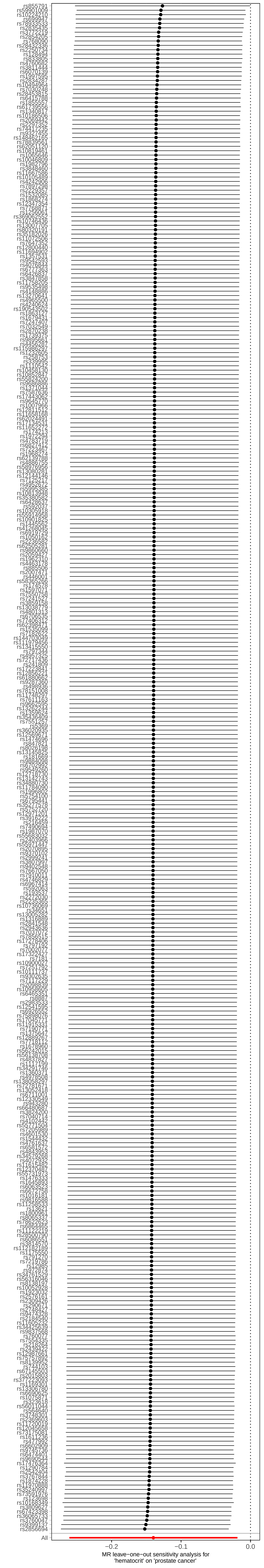

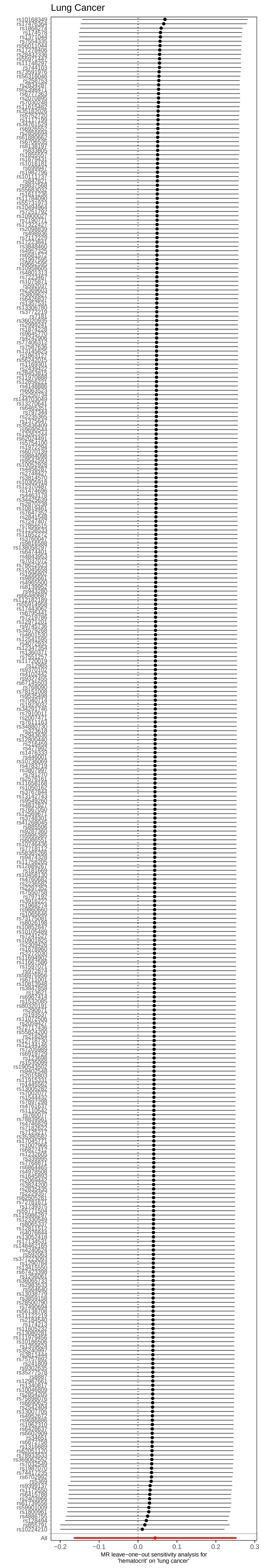
Lung Cancer
All
−0.2
−0.1
0.0
0.1
0.2
0.3
MR leave−one−out sensitivity analysis for
'hematocrit' on 'lung cancer'

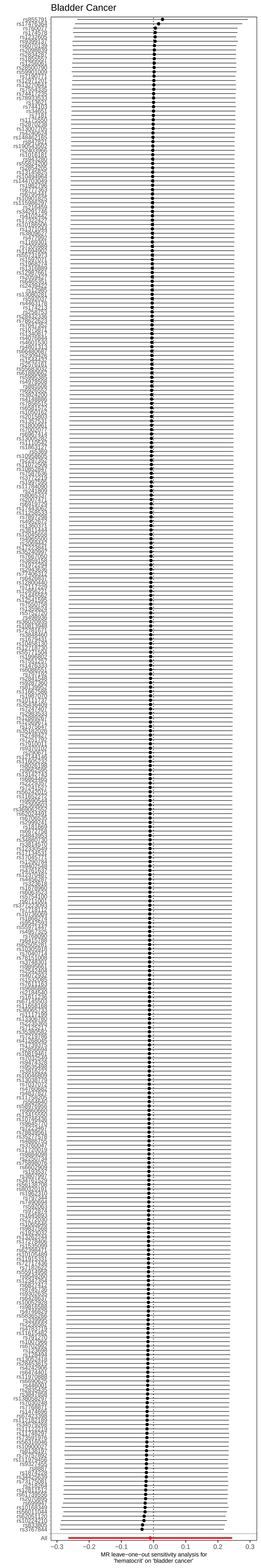
Bladder Cancer
All
−0.3
−0.2
−0.1
0.0
0.1
0.2
0.3
MR leave−one−out sensitivity analysis for
'hematocrit' on 'bladder cancer'

Renal Cancer

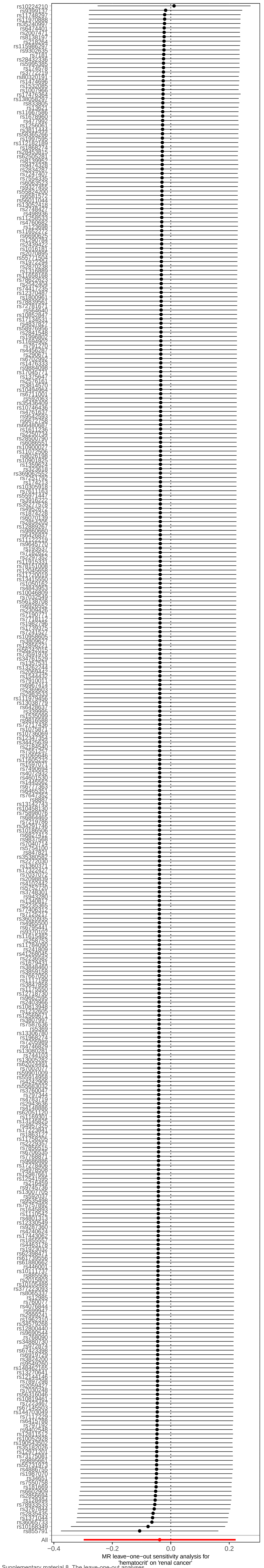

Supplementary material 8. The leave-one-out analyses.
