# Supplementary material for: Correlation between hemoglobin and the risk of common malignant tumors: a 1999–2020 retrospective analysis and causal association analysis
Source: BMC Cancer. 2024 Jun 21;24:755. doi: 10.1186/s12885-024-12495-0 (PMC11193233; doi:10.1186/s12885-024-12495-0)
Supplement: Supplementary file 9 — Supplementary Material 9 [file 12885_2024_12495_MOESM9_ESM.pdf]

Supplementary material 9. Outcome cohorts for repeated MRAs for specific cancers.

**System**	**Cancer type**	**Sample size (control vs. case)**	**Data source**
Circulatory system	Myeloid leukemia	372016 vs. 462	ieu-b-4958
Urinary system	Bladder cancer	372016 vs. 1279	ieu-b-4874
	Renal cancer	461896 vs. 1114	ukb-b-1316
Others	Melanoma	372016 vs. 3751	ieu-b-4969

Notes: The data are available from https://gwas.mrcieu.ac.uk/datasets.
