# Supplementary material for: Correlation between hemoglobin and the risk of common malignant tumors: a 1999–2020 retrospective analysis and causal association analysis
Source: BMC Cancer. 2024 Jun 21;24:755. doi: 10.1186/s12885-024-12495-0 (PMC11193233; doi:10.1186/s12885-024-12495-0)
Supplement: Supplementary file 10 — Supplementary Material 10 [file 12885_2024_12495_MOESM10_ESM.pdf]

Supplementary material 10. The Egger regression analysis for detecting horizontal pleiotropy of repeated MRAs.

Exposure	Cancer outcome	Egger intercept	SE	*P* value
Hemoglobin	Bladder cancer	<0.001	<0.001	0.601
Hemoglobin	Melanoma	<0.001	<0.001	0.919
Hemoglobin	Myeloid leukaemia	<0.001	<0.001	0.723
Hemoglobin	Renal cancer	<0.001	<0.001	0.182
