# Supplementary material for: Correlation between hemoglobin and the risk of common malignant tumors: a 1999–2020 retrospective analysis and causal association analysis
Source: BMC Cancer. 2024 Jun 21;24:755. doi: 10.1186/s12885-024-12495-0 (PMC11193233; doi:10.1186/s12885-024-12495-0)
Supplement: Supplementary file 11 — Supplementary Material 11 [file 12885_2024_12495_MOESM11_ESM.pdf]

Supplementary material 11. The Cochran Q test for detecting heterogeneity of repeated MRAs.

Method	Exposure	Outcome	Cochran's Q value	*P* value
Inverse variance weighted	Hemoglobin	Bladder cancer	351.353	0.381
Inverse variance weighted	Hemoglobin	Melanoma	378.060	0.106
Inverse variance weighted	Hemoglobin	Myeloid leukaemia	321.032	0.797
Inverse variance weighted	Hemoglobin	Renal cancer	182.644	0.350
