# Supplementary figures and images for: Correlation between hemoglobin and the risk of common malignant tumors: a 1999–2020 retrospective analysis and causal association analysis
Source: BMC Cancer. 2024 Jun 21;24:755. doi: 10.1186/s12885-024-12495-0 (PMC11193233; doi:10.1186/s12885-024-12495-0)

# Myeloid Leukaemia

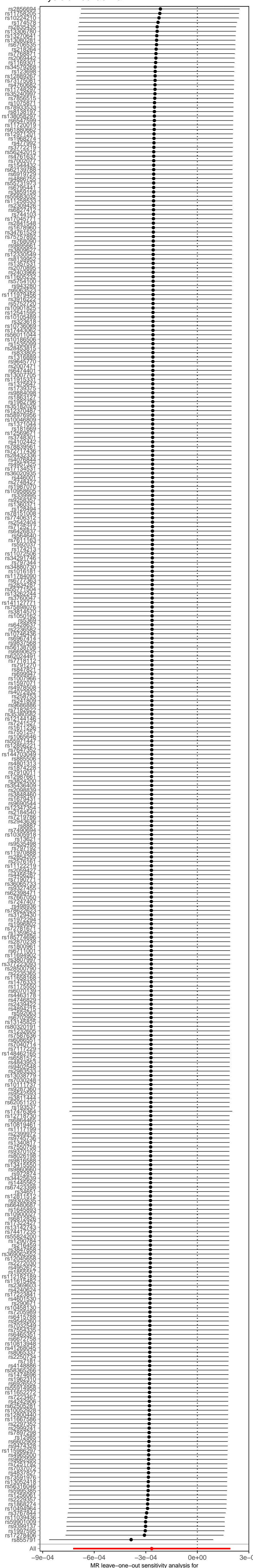

# Melanoma

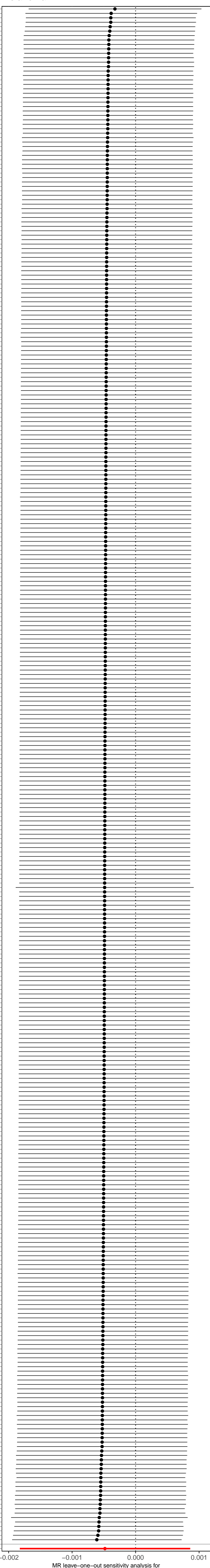

# Bladder Cancer

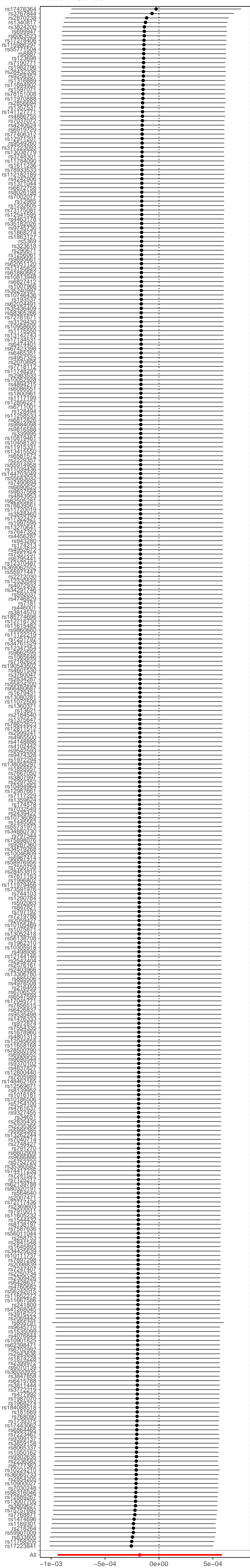

# Renal Cancer

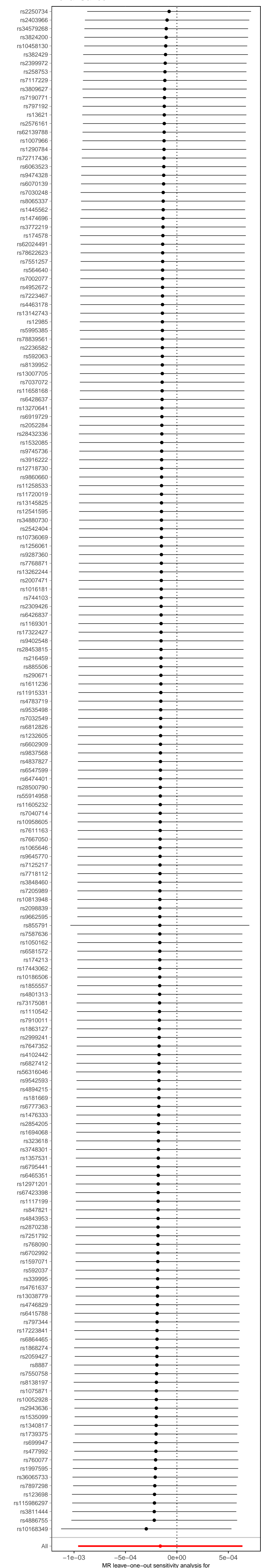

Supplement: Supplementary file 12 — Supplementary Material 12 [file 12885_2024_12495_MOESM12_ESM.pdf]
